# Snakebites in Pediatric Patients in Kahramanmaraş: Is Pro-brain Natriuretic Peptide a Prognostic Biomarker for Snakebites?

**DOI:** 10.7759/cureus.21570

**Published:** 2022-01-24

**Authors:** Sevcan İpek, Sukru Gungor, Ufuk U Güllü, Tahir Dalkıran, Mehmet Mercan, Şeyma Demiray, Yunus Gürbüz

**Affiliations:** 1 Pediatric Critical Care, Kahramanmaraş Sütçü İmam University Faculty of Medicine, Kahramanmaraş, TUR; 2 Pediatric Gastroenterology, Kahramanmaraş Sütçü İmam University Faculty of Medicine, Kahramanmaraş, TUR; 3 Pediatric Cardiology, Kahramanmaraş Sütçü İmam University Faculty of Medicine, Kahramanmaraş, TUR; 4 Department of Pediatric Intensive Care Unit, Kahramanmaraş Necip Fazıl City Hospital, Kahramanmaraş, TUR; 5 Department of Pediatrics, Kahramanmaraş Necip Fazıl City Hospital, Kahramanmaraş, TUR; 6 Pediatrics, Kahramanmaraş Sütçü İmam University Faculty of Medicine, Kahramanmaraş, TUR; 7 Pediatric Intensive Care Unit, Kahramanmaraş Sütçü İmam University Health Practice and Research Hospital, Kahramanmaraş, TUR

**Keywords:** snakebite, probnp, pediatric, complex regional pain syndrome, antivenom

## Abstract

Background: Snake envenomations are a serious cause of mortality and morbidity in the world.

Aims: This study was conducted to investigate snake bites in pediatric patients in Kahramanmaraş and to determine whether pro-brain natriuretic peptide (proBNP) has a prognostic value in these patients.

Methods: Pediatric patients aged <18 years who presented to the pediatric emergency department with snakebites were reviewed retrospectively. The demographical, clinical, laboratory, treatments, and outcomes data were collected from their medical records. Stage 0 and 1 envenomation was considered as a non-serious complication and stage 2 and 3 envenomation was considered as a serious complication.

Results: A total of 32 pediatric patients, six females and 26 males, between 2016 and 2021, were included in the study. The mean age was 12.52±3.28 years. There were seven patients without serious complications and 25 patients with serious complications. The best cutoff point for proBNP to predict serious complications was found to be ≥272.5 ng∙L^-1^ (sensitivity, 83.3%; specificity, 100%, *p*=0.011). We also detected complex regional pain syndrome in one of our patients.

Conclusions: In this study, proBNP was shown to be predictive of a poor outcome of snakebites. Moreover, complex regional pain syndrome, which is rarely reported in the literature, should be kept in mind during the long-term follow-up of snakebites.

## Introduction

According to statistics from the World Health Organization, 5.4 million snakebites occur every year worldwide, and 1.8 to 2.7 million of these cases are caused by venomous snakes. Every year, 81,000-138,000 deaths occur due to snakebites, and permanent disability, especially amputation, occurs approximately three times as often [1]. The Viperidae family is responsible for most snakebite envenomation cases in Turkey, and the most commonly involved members of this family are Vipera ammodytes, V. xanthine, and V. labetina [2]. Kahramanmaraş, Turkey, has a variable climate structure due to its geographical location. It has an average elevation of 568 m, and the northern parts of the city are particularly mountainous. The southern parts of the city display a Mediterranean climate, and the northern parts display a continental climate. This makes Kahramanmaraş rich in terms of plant and animal diversity [3].

Recently, studies with brain natriuretic peptide (BNP) have started to gain importance in terms of human physiology [4]. BNP is widely used as a biomarker in heart diseases. BNP is mainly released from left ventricular myocytes as a 134-amino acid peptide preproBNP in response to stretching the ventricle under volume load and pressure. BNP is also synthesized and released by hormonal and sympathetic stimuli. The 26-amino acid signal peptide is then cleaved, and the 108-amino acid proBNP is formed. It is further cleaved into the active 32-amino acid BNP and the 76-amino acid inactivated NT-proBNP. BNP is involved in natriuresis, diuresis, vasodilation, and inhibition of renin-angiotensin aldosterone (RAA). BNP binds to natriuretic peptide receptor A found in kidney, adrenal gland, lung, terminal ileum, aorta, and adipose tissue. With receptor activation, the intracellular secondary messenger increases in cyclic guanosine monophosphate, resulting in vasodilation, natriuresis, aldosterone inhibition, and lipolysis [5].

Depending on the type of snake and the amount of venom ingected, snakebites can present a clinical spectrum ranging from local symptoms to multiorgan failure. The most important affected system is the circulatory system. Researchers have proposed evaluation tools such as troponin-I and ECG to evaluate the effect of snake venom on the heart [6]. However, we could not find any study evaluating proBNP in snakebites. In addition, one study emphasized that proBNP has predictive value for morbidity in scorpion envenomation [7]. Considering this information, we think proBNP can be used as a prognostic marker in snakebites. Therefore, in our study, we aimed to share our clinical experience in pediatric patients presenting with snakebites and to evaluate the prognostic importance of proBNP in snakebites.

## Materials and methods

Patients

Patients aged <18 years presenting at Kahramanmaraş Sutcu Imam University (KSU) Faculty of Medicine and Kahramanmaraş Necip Fazıl City Hospital with snakebites between 2016 and 2021 were retrospectively included, and their demographic characteristics, clinical findings, laboratory data, treatments, and outcomes were collected from their medical records. Additionally, the patients were contacted by phone, and information was collected about their current status.

Ethical approval

This study was conducted in accordance with the principles of the Declaration of Helsinki. Ethical approval was obtained from the ethics committee of KSU with the approval number 2020/09-01.

Snakebite staging

Cases of envenomation due to snakebites were standardized in 4 stages: Stage 0: bite site only without local or systemic findings (dry bite), Stage 1: systemic symptoms absent but minimal tissue swelling and normal laboratory findings, Stage 2: advancing swelling, mild systemic symptoms such as pain, ecchymosis, and some abnormal laboratory results, Stage 3: further advanced swelling, necrosis, bullous lesion, extreme pain, necrosis, and severe systemic findings such as coagulopathy and organ failure [8].

In the envenomation section of Davidson's Principles and Practice of Medicine, it is stated that dry bites do not require antivenom, the onset of the first symptoms and signs of poisoning varies according to the patient and the animal. Also in some cases, poisoning symptoms can be seen from a few minutes to 24 hours after the bite, and some poisonings can only be detected by laboratory tests [9]. Therefore, dry bites were also included in the study.

Stage 0 and 1 cases were evaluated as the group without serious complications, and Stage 2 and 3 cases were evaluated as the group with serious complications.

Measurement of proBNP

Serum proBNP levels were determined by using The AQT90 FLEX analyzer (Radiometer Medical, Copenhagen, Denmark) and the cutoff value was set at 133 ng/L.

Treatment protocol

The patients with severe snakebite envenomations were treated with polyvalent snake antivenom (polyvalent snake antivenom, thsk.tglab@saglık.gov.tr) produced by the Public Health Institution of the Republic of Turkey. This antivenom comes in 5 ml ampoules of 10 ml in a box (5 ml ampoule one vial). Ten millilitres of antivenom contains a minimum of 500 LD50 Montivipera Xanthina, 500 LD Macrovipera Lebetina, and 500 LD Vipera Ammodytes equine-sourced globulins that neutralize snake venom. Before the antivenom was given, premedication with antihistamines and steroids was applied to prevent an allergic reaction. The tetanus vaccine was routinely administered to the patients.

Definitions based on clinical and laboratory data

The site of the bite was categorized into four regions: upper extremity, lower extremity, head-neck, and trunk. Rhabdomyolysis was defined when the serum creatine kinase (CK) level was >1,000 U∙L-1 or at least five times the upper normal limit [10]. The coagulation disorder was defined when international normalized ratio (INR) >1.2, platelet <100 x 109⋅mL-1, fibrinogen <150 mg∙dL-1, D-dimer >0.5 mg∙mL-1, prothrombin time (PT) ≠11.6-13.2 sec, and activated partial thromboplastin time (aPTT) ≠30.1-36.9 sec [11]. Acute kidney injury (AKI) was defined as follows: stage 1 serum creatinine increases by 26 mmol∙L-1 or 1.5-1.9 times the reference creatinine value, two to 2.9 times the stage 2 reference creatinine value, and stage 3 AKI three times the reference creatinine value or a creatinine increase >354 mmol∙L-1 [12].

Exclusion criteria

Patients with heart disease, kidney disease, pneumonia or central nervous system diseases were excluded.

Statistical analyses

The Statistical Package for Social Sciences (SPSS) version 22 (IBM Corp., Armonk, NY, USA) software package was used for statistical analysis. Study variables are presented as numbers (n) - percentages (%) or means ± standard deviations. The normal distribution of variables was tested using the Kolmogorov-Smirnov test. Normally distributed parameters were evaluated by one-way analysis of variance or Student's t-test; nonnormally distributed numerical variables were evaluated by the Kruskal-Wallis or Mann-Whitney U test. Student's t-test, Mann-Whitney U test, or the chi-square test was used to evaluate the statistical significance. To predict morbidity from snakebites, we analyzed the best cutoff point of proBNP via the recipient operator characteristic (ROC) curve.

## Results

All of the patients presenting with snakebites were found to be healthy before the bite according to their medical records. When the patients applied to the emergency department, they or their parents said that they did not have any disease and that they were healthy until that day. No congenital or acquired heart disease was detected in echocardiography of patients with stage 2 and stage 3 envenomation. They did not have any prior health problems, such as heart disease, kidney disease, or central nervous system disease. Therefore, all patients were included. Their demographic and clinical characteristics are shown in Table 1.

**Table 1:** Demographical and clinical parameters

Variable	n (%)
Age (years) (mean±SD)	12.52±3.28
Sex	
Male	26 (81.2%)
Female	6 (18.8%)
Grading	
0	3 (9.4%)
1	4 (12.5%)
2	16 (50%)
3	9 (28.1%)
Site of bite	
Upper extremity	16 (50%)
Lower extremity	15 (46.9%)
Head-neck	1 (3.1%)
Trunk	0 (0%)
Season	
Autumn	7 (21.9%)
Spring	5 (15.6%)
Summer	20 (62.5%)
Winter	0 (0%)
Symptoms	
Pain	32 (100%)
Swelling	28 (87.5%)
Erythema	28 (87.5%)
Ecchymosis	18 (56.3%)
Necrosis	6 (18.8%)
Nausea	7 (36.8%)
Angioedema	1 (3.1%)
Rhabdomyolysis	3 (11.5%)
Coagulation disorder	8 (25%)
Renal injury	1 (3.1%))
Bleeding	2 (6.3%)
Amputation	2 (6.3%)
Fasciotomy	7 (21.9%)
Neurotoxicity	1 (3.1%)
Compartment syndrome	8 (25%)
Antivenom (vial) (mean (min-max))	5.3 (2-16)

A total of 32 pediatric cases (six female, 26 male) of viper snakebites between 2016 and 2021 were included in this study. The mean age of the patients was 12.52±3.28 years. We found that the rate of boys who presented with snakebites was approximately four times higher than that of girls. The type of snake was determined according to the statements of the patients and their relatives or according to the photographs of the snakes. Cases of envenomation were mostly at stage 2 (16 cases [50%]). There were seven patients in the group without serious complications and 25 patients in the group with serious complications. We found that the rate of bites inflicted to the lower and upper extremities was high (15 cases [46.87%] and 16 cases [50%], respectively). We found that snakebite cases were most common in summer, with 20 cases (62.5%), and that July had the highest number of cases, with eight (21.9%). In addition, 18 (58.1%) patients were admitted between 5 p.m. and 8 p.m. The earliest case reported in the calendar year was seen on the 25th of April, and the last case was seen on the 28th of October. Pain, swelling, and erythema were the most common complaints (32 cases [100%], 28 cases [87.5%], and 28 cases [87.5%], respectively). Tissue necrosis was seen in six cases (18.8%), angioedema in one (3.1%), coagulation disorder in eight (25%), renal dysfunction (Stage 1 AKI) in one (3.1%), bleeding in two (6.3%), amputation in two (6.3%), compartment syndrome in eight (25%), and fasciotomy in seven (21.9%). Rhabdomyolysis was not observed in any patients, but CK elevation four times the normal value was detected in three cases (11.5%). Although encephalopathy, seizure, aphasia, and focal neurological deficit were not observed in our patients, a 12-year-old male patient who was bitten by a snake had numbness in the hands and feet and a bitter taste in the mouth. None of the patients died.

**Figure 1:**
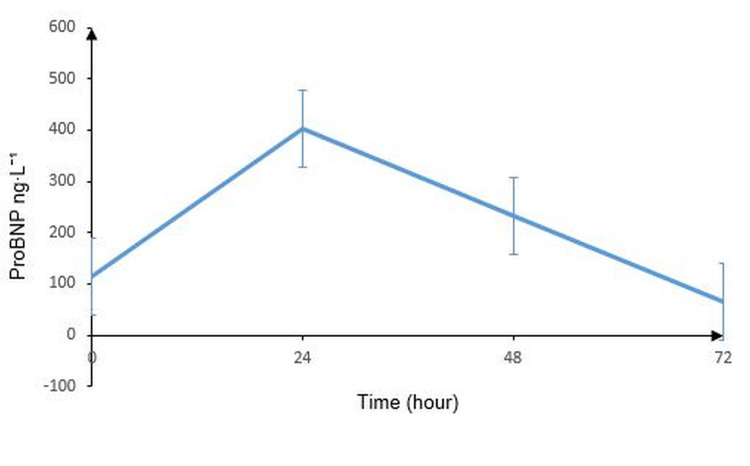
Mean proBNP values of patients presenting with snakebites. ProBNP, pro-brain natriuretic peptide

When the patients were evaluated according to their laboratory data at admission and for the following three days, there was no significant difference between these times for the parameters of white blood cells (WBCs), eosinophils (EOs), platelets (PLTs), plateletcrit (PCT), mean platelet volume (MPV), creatinine kinase (CK), creatinine kinase-MB (CK-MB), INR, or D-dimer. Red blood cell distribution (RDW, p=0.032), proBNP (p=0.002) (Figure 1), and neutrophil-lymphocyte ratio (NLR, p=0.017) were significantly higher at 24 hours and fibrinogen at 72 hours (p=0.041) (Table 2).

**Table 2:** Comparison of laboratory data according to days of patients presenting with snakebite * One-way ANOVA-Scheffe alfa (0.05); CK-MB, creatine kinase MB; ProBNP, pro-brain natriuretic peptide; INR, international normalized ratio

Parameters	At initial	24^th^ hour	48^th^ hour		72^nd^ hour	p^*^
White blood cell (10^3^⋅mL^-1^)	10,220±3,421	12,240±6,219	12,360±7,219	9,920±6,160		0.361
Eosinophil (10^3^⋅mL^-1^)	150±155	100±195	110±117	170±0,183		0.519
Platelets (10^9^⋅mL^-1^)	226,590±72,295	239,290±63,188	211,880±65,73	251,460±104,860		0.478
Plateletcrit (%)	0.299±0.345	0.218±0.060	0.201±0.055	0.236±0.092		0.400
Mean platelet volume (fL)	9.80±1.03	9.97±1.39	10.15±1.35	10.00±1.10		0.816
Red blood cell distribution (%)	13.20±1.39	16.58±8.82	12.96±1.06	13.01±1.05		0.032
Creatinine kinase (U∙L^-1^)	173.51±158.87	201.14±218.88	201.24±218.89	97.87±74.88		0.570
CK-MB (ug∙L^-1^)	3.06±2.85	3.89±3.57	1.72±1.13	2.42±1.45		0.431
ProBNP (ng∙L^-1^)	113.56±94.05	401.83±335.74	233.44±199.27	66.00±71.00		0.002
INR	1.08±0.109	1.16±0.191	1.12±0.269	1.09±0.203		0.484
Fibrinogen (mg∙dL^-1^)	209.00±62.60	228.81±80.38	311.31±114.62	320.25±121.63		0.017
D-Dimer (mg∙L^-1^)	4.95±6.95	2.98±3.69	1.33±1.93	3.17±5.76		0.508
Neutrophil-lymphocyte ratio	5.66±6.19	13.74±18.50	8.14±9.26	4.27±4.59		0.041

Comparing the laboratory values of patients with and without serious complications at 24 hours after the snakebite, we found no statistically significant difference in terms of WBC, EO, PLT, MPV, RDW, CK, CK-MB, INR, fibrinogen, D-dimer, or NLR (p=0.182, p=0.400, p=0.272, p=0.526, p=0.282, p=0.944, p=0.525, p=0.081, p=0.776, p=0.062, p=0.642, respectively). However, proBNP was significantly higher in the group with serious complications (p=0.025). Additionally, we found a significantly lower PCT value in the group with serious complications (p=0.018) (Table 3).

**Table 3:** Comparison of laboratory data after 24 hours for patients with and without serious complications after snakebite * Independent student t-test; CK-MB, creatine kinase MB; ProBNP, pro-brain natriuretic peptide; INR, international normalized ratio

Variable	Serious complication		p^*^
	No (n=7) (mean±SD)	Yes (n=25) (mean±SD)	
White blood cell (10^3^⋅mL^-1^)	9,760±3,730	13,180±8,040	0.182
Eosinophil (10^3^⋅mL^-1^)	61±92	131±244	0.400
Platelets (10^9^⋅mL^-1^)	256,400±52,130	227,070±69,260	0.272
Plateletcrit (%)	0.252±0.051	0.195±0.055	0.018
Mean platelet volume (fL)	10.19±0.813	9.80±1.728	0.526
Red blood cell distribution (%)	18.89±11.05	14.81±6.58	0.282
Creatinine kinase (U∙L^-1^)	206.16±192.53	197.37±249.92	0.944
CK-MB (ug∙L^-1^)	3.37±2.22	4.74±5.308	0.525
ProBNP (ng∙L^-1^)	124.40±96.34	466±267.82	0.025
INR	1.08±0.062	1.22±0.235	0.081
Fibrinogen (mg∙dL^-1^)	237.00±58.93	222.00±100.11	0.776
D-Dimer (mg∙L^-1^)	0.39±0.22	4.28±3.97	0.062
Neutrophil-lymphocyte ratio	15.26±21.07	25.36±64.99	0.642

We found the best cutoff point of proBNP to be ≥272.5 ng∙L-1 to predict serious complications at 24 hours in patients presenting with snakebites. According to this cutoff point, proBNP has a sensitivity of 83.3% and a specificity of 100% for predicting serious complications (p=0.011). We determined the best cutoff point of PCT to predict serious complications to be ≤0.235. According to this cutoff point, it has a sensitivity of 60% and a specificity of 85.7% in predicting serious complications (Table 4).

**Table 4:** Determination of proBNP cut-off point to predict serious complication in patients with snakebite ProBNP, pro-brain natriuretic peptide

Variable	Cut-off	AUC	Sensitivity	Specificity	Asymptotic 95% Confidence Interval	p
ProBNP (ng∙L^-1^)	≥272.5	0.967	0.833	1	0.868-1	0.011
Plateletcrit (%)	≤0.235	0.743	0.600	0.857	0.539-0.947	0.046

Antivenom was given to 10 (31.25%) patients. The mean amount of antivenom administered was 5.3 vials (min: two, max: 16). No reaction against antivenom developed in any of the patients.

**Figure 2:**
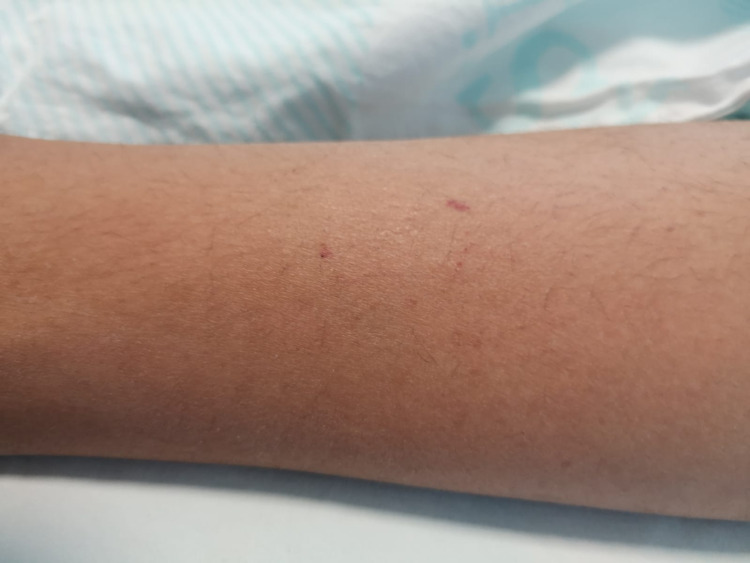
Dry bite and tooth marks in a snakebite patient.

**Figure 3:**
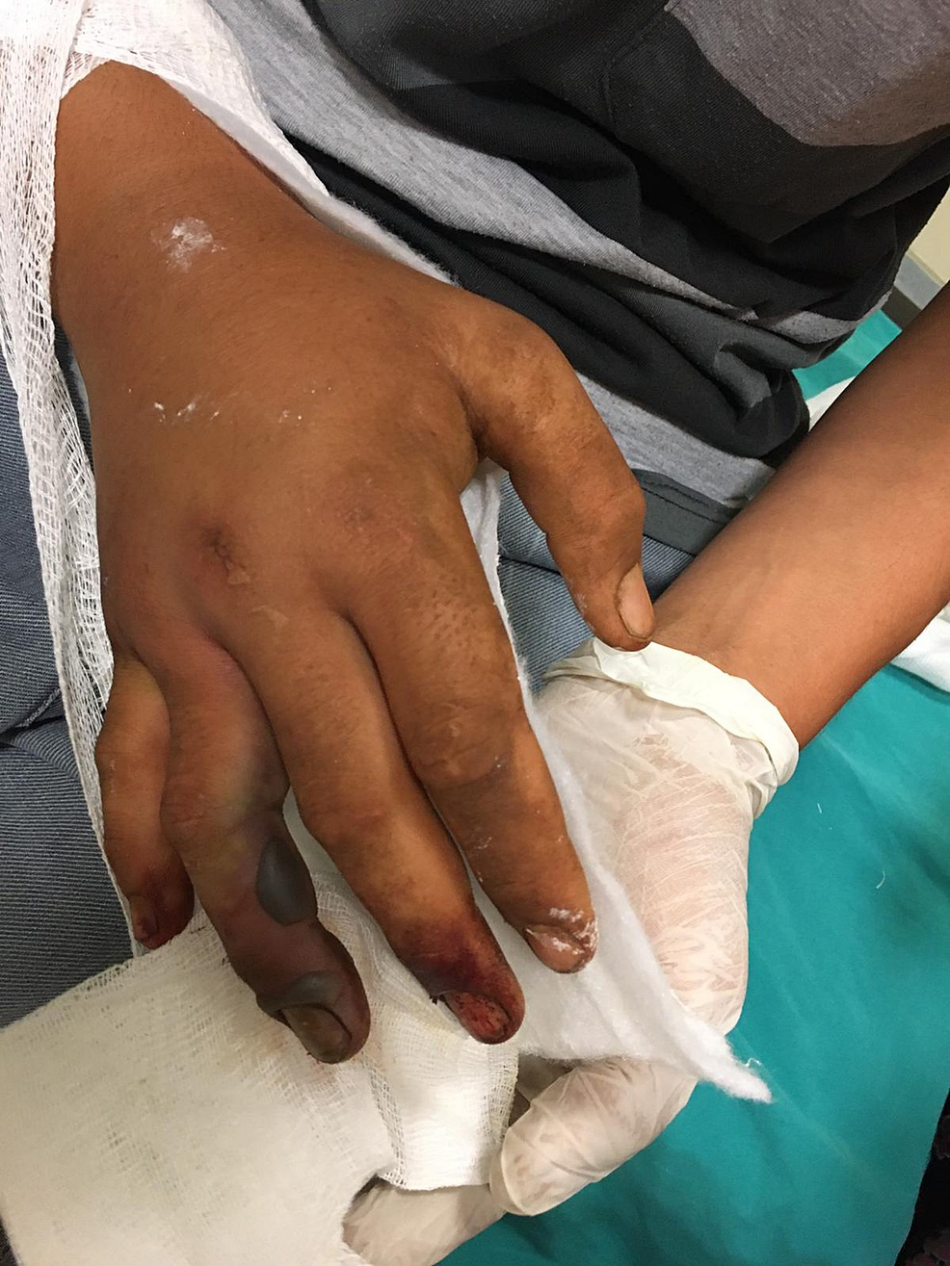
Necrosis and bullae developed in the distal phalanx of patient 22.

**Figure 4:**
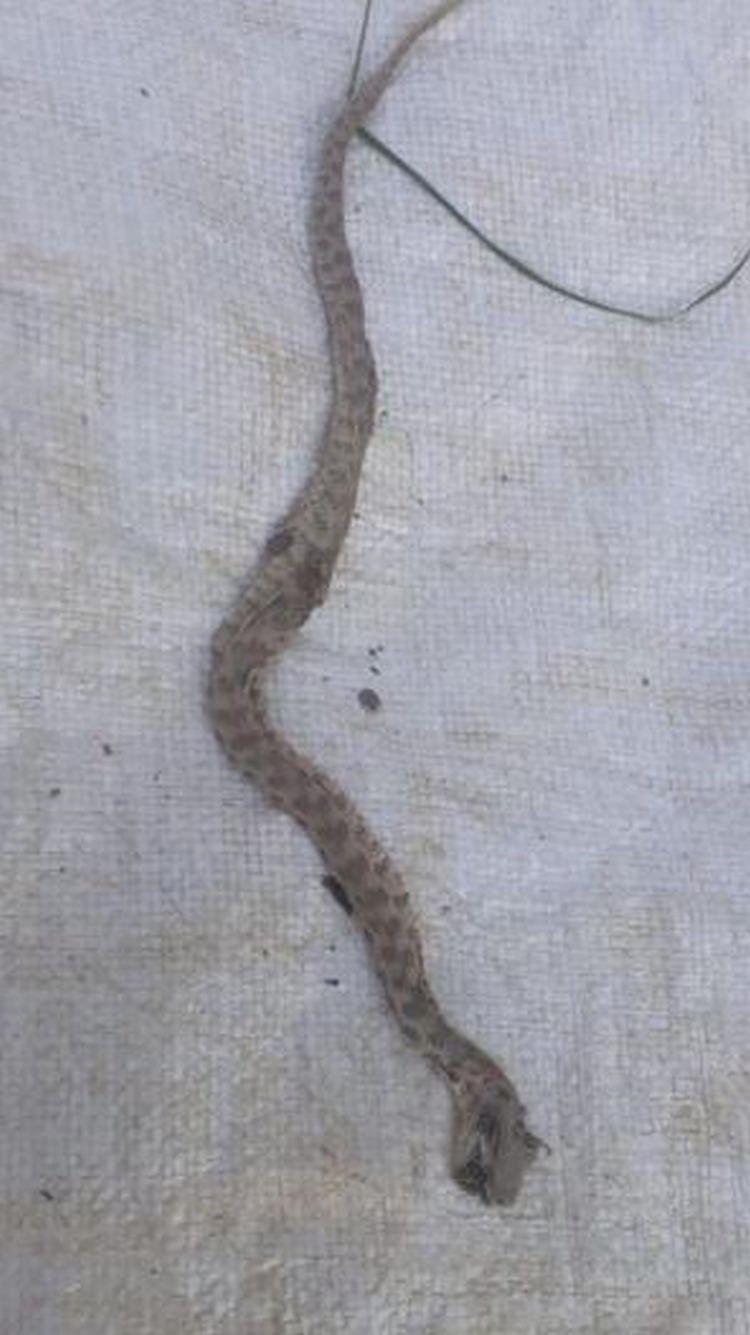
The viper snake killed by the relatives of patient 22.

**Figure 5:**
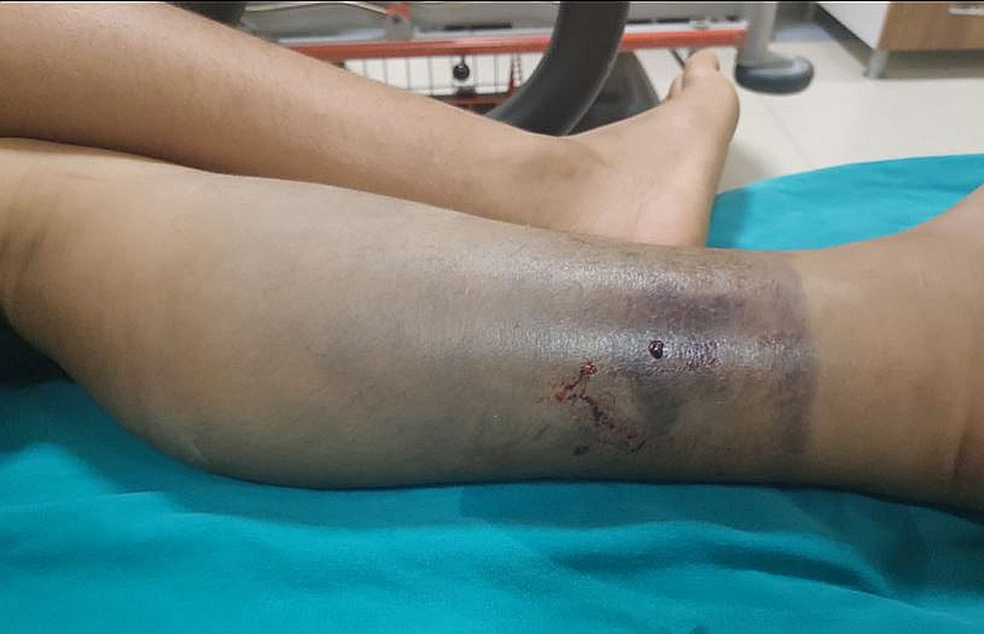
A patient who was bitten on the leg showed widespread swelling and ecchymosis on the leg.

**Figure 6:**
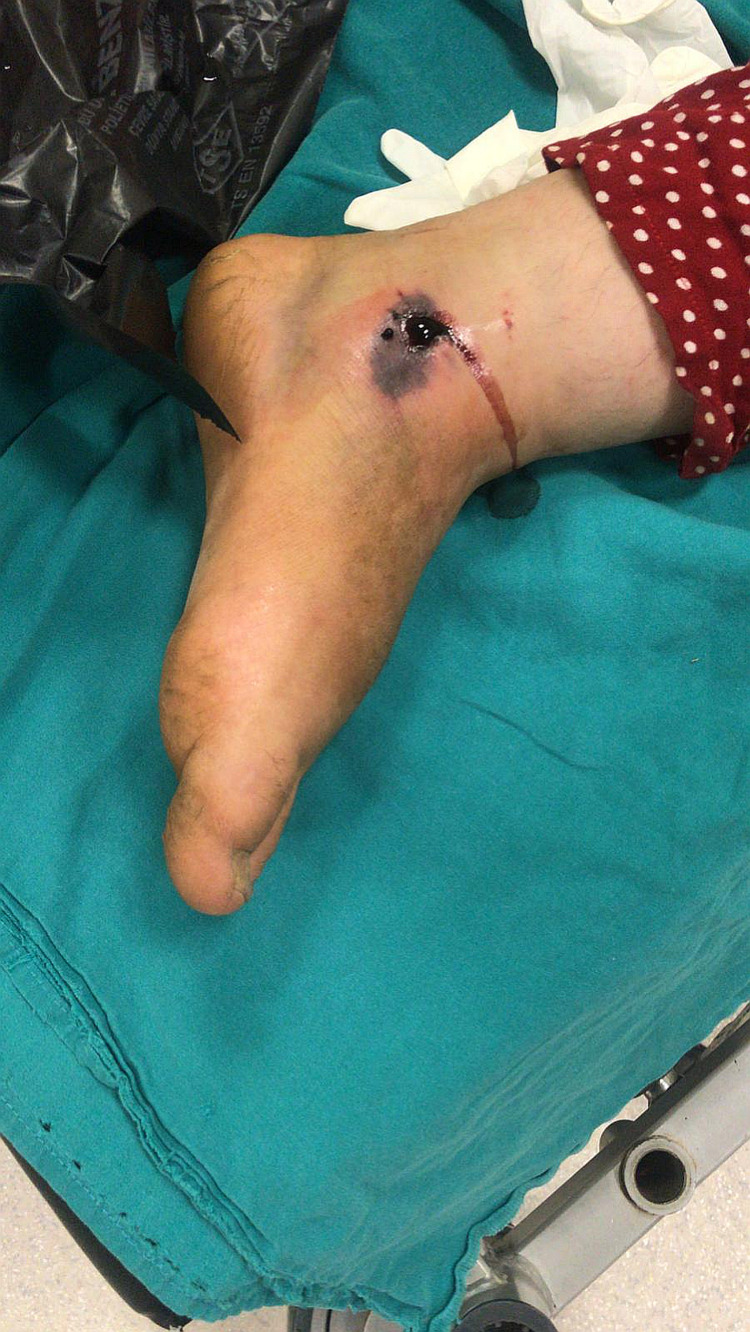
A patient who was bitten on the leg showed necrosis and bullae.

The pictures show the tissue damage observed in the patients and the snake killed by the relatives of the patient. Accordingly, fang marks are seen in the patient with a dry bite in Figure 2. In Figure 3, necrosis and bullae developed in the distal phalanx of patient 22, and the snake that bit the patient and was killed by the patient's relatives can be seen in Figure 4. In Figure 5, a patient who was bitten on the leg shows widespread swelling and ecchymosis on the leg. In Figure 6, a patient who was bitten on the leg shows necrosis and bullae.

**Figure 7:**
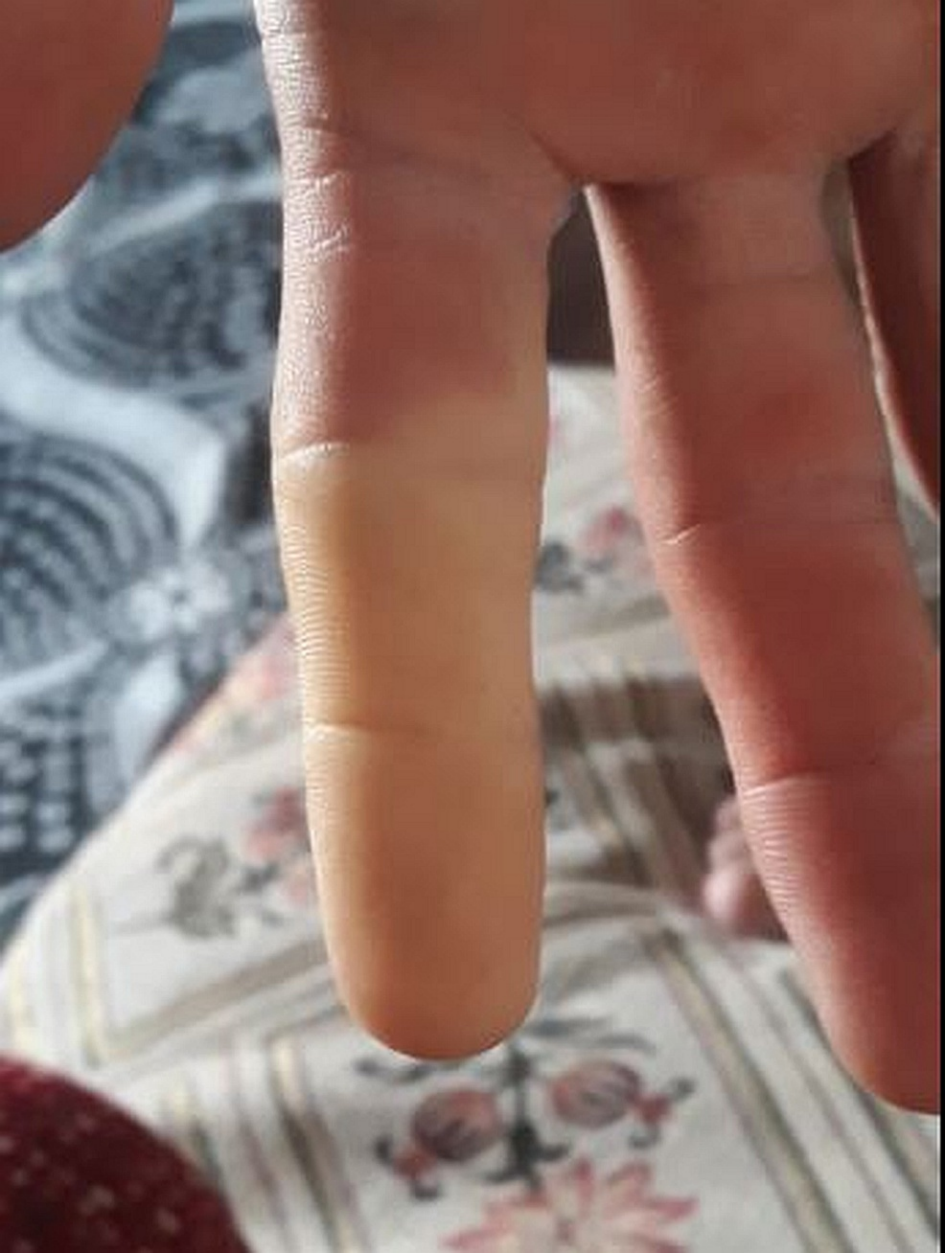
A patient who developed complex regional pain syndrome after exposure to cold air 6 months after the snakebite.

Among our patients, compartment syndrome developed in patient 14, who was bitten on the finger in July, but fasciotomy was not performed. He received 14 vials of antivenom, his hand healed, and he was discharged. However, after six months, extreme pain and pallor developed in his finger after exposure to cold weather in January (Figure 7). The patient was diagnosed with complex regional pain syndrome.

## Discussion

In our study, we presented our clinical experience with snakebites in pediatric patients in Kahramanmaraş. Studies have reported that snakebites are most common in June [13]. However, in our study, snakebites were detected mostly in July. Since the ratio of body surface area to body weight is higher in children than adults, the amount of poison they are exposed to is higher, and they experience the toxic effects of snake venom more severely [14]. In terms of the local tissue damage, only pain can be felt, but serious damage such as tissue necrosis can also be seen. Systemic effects may include nausea, vomiting, hypotension, tachycardia, dyspnea, dry mouth, paresthesia, generalized edema, and cyanosis. Life-threatening coagulopathy, neurotoxicity, myotoxicity, and acute kidney injury can also occur. Previous studies of snakebites in Turkey have reported pain, compartment syndrome, local swelling, ecchymosis, renal failure, rhabdomyolysis, hemolysis, and convulsions [15]. Pain and swelling were the most common symptoms in our pediatric patients. We also observed that compartment syndrome developed in 25% of the patients, fasciotomy was performed in 21.9%, tissue necrosis developed in 18.8%, and unfortunately, 6.3% resulted in amputation. Although antivenom was administered to a patient who applied six hours after the bite, unfortunately, amputation was performed. In this patient, the family had previously applied a tight tourniquet. Another patient who required amputation was administered antivenom two hours after the bite, but a tight tourniquet had been applied to this patient by the family. Substances contained in snake venom may cause tissue necrosis, and inappropriate and unnecessary applications such as tight tourniquets, pressure dressings, suction, local incisions, bleeding, and the use of ice may further disrupt tissue circulation and facilitate the development of necrosis [16]. Therefore, providing appropriate first aid for snakebites and informing the public in this regard is important.

Snake venom can have a hematotoxic effect with procoagulant, anticoagulant, fibrinolytic, and platelet activation or inactivation properties [17]. One study in Sri Lanka showed that hemostatic dysfunction developed in 6% of patients after hump-nosed viper bites [18]. Another study in Turkey detected hematological abnormalities in 15% of the patients [19]. Coagulation disorder was detected in 25% of our patients.

Timely administration of antivenom can accelerate recovery and reduce the likelihood of long-term injuries. While antivenom therapy is life-saving, attention should be given to its side effects, such as allergic reactions and serum sickness [20]. In our study, the fact that no allergic reaction developed in patients treated with antivenom may be due to the premedication with steroids and antihistamines.

Cardiac toxicity of the venom in snakebites has rarely been reported because snakebites usually occur in rural areas where access to health centers is difficult. Phospholipase A2 (PLA2) found in the venom of venomous snakes is cardiotoxic and it shows this effect by causing hypotension together with the other snake venom components [21]. The Elapidae snake venom contains ANP/BNP natriuretic peptides and CNP natriuretic peptides are found in Viperidae [22]. It has been reported that a Northern Sri Lanka saw-scaled viper bite caused acute myocardial infarction in one patient, but the patient recovered with appropriate treatment [6]. BNP is used extensively in the diagnosis and treatment of heart diseases. BNP exerts vasodilator, diuretic, and natriuretic effects by antagonizing the sympathetic nervous system and RAA system. Mechanical stress, ischemia, hypoxia, and neurohumoral factors cause BNP synthesis and secretion. However, the exact mechanisms involved in its regulation are not clear. In animal experiments, it increased in as little as 10 minutes after arrhythmia [23]. A decrease in BNP in cardiovascular diseases indicates an improvement in clinical symptoms, and there is a positive correlation between the risk of death and increased BNP. In some studies, it has been reported that natriuretic peptides are prognostic in patients with acute coronary syndrome. In animal and human studies, it has been stated that when hypoxic pulmonary vasoconstriction is created, BNP increases and causes pulmonary vasodilation. It has been reported that high BNP is associated with an increased risk of death in patients with acute respiratory distress syndrome (ARDS) [24]. Güllü et al. studied cases of envenomation due to scorpion bites and showed proBNP to have a good predictive value for a poor outcome [7]. In this study, we determined a cutoff point for serum proBNP levels at 24 hours after snakebite to detect patients who can develop serious complications due to snakebites (sensitivity 83.3%, specificity 100%, p=0.011). To the best of our knowledge, this predictive data for proBNP in snakebites is presented for the first time.

Platelet indices such as PCT and platelet distribution width (PDW), which are inexpensive and can be measured almost everywhere, are being widely researched as new biomarkers for the diagnosis and prognosis of acute and chronic diseases. PCT and PDW are associated with inflammation [25]. Çelik et al. examined scorpion stings in children and showed that increased platelet and PCT values were predictive of serious clinical conditions [26]. In another study, an increased PDW and PDW to lymphocyte ratio showed a poor prognosis in snakebites [19]. In our study, however, PCT was found to be lower in the group with serious complications and was predictive of a poor outcome.

Complex regional pain syndrome (CRPS) is a disease that often develops after trauma and is characterized by pain, edema, skin changes, atrophy, osteoporosis, and contractures in the extremities. Its pathophysiology remains unclear, and it has no specific symptoms or pathognomonic findings. Significant pain in the affected limb, even with a light touch, cold and hot sensation, or red or pale discoloration, indicates sympathetic dysfunction. However, there is no specific laboratory method to diagnose this [27]. Snake venom PLA2 shows myotoxic activity and evokes inflammatory and nociceptive responses [28]. Subcutaneous injection of PLA2s has been shown to induce hyperalgesia in rats [29]. Ergan et al. detected complex partial pain syndrome after snakebite in a 66-year-old patient [30]. We also described CRPS that developed after snakebite in one of our patients, which has rarely been described in the literature. Doppler USG was performed and evaluated as normal. The patient was diagnosed with complex regional pain syndrome and was treated symptomatically. This syndrome should be kept in mind among the complications after snakebites to prevent the possible loss of serious function and to achieve pain control by early treatment.

The main limitations of our study are its retrospective design and the small number of patients.

## Conclusions

In venomous snakebites, timely and appropriate treatment is important to reduce mortality and morbidity. Although our study showed that proBNP (≥272.5 ng∙L^-1^) is predictive of a poor outcome of snakebites, more comprehensive and prospective studies with different snake species are necessary before its clinical use. In addition, complex regional pain syndrome, which is rarely reported in the literature, should be kept in mind to reduce morbidity in the long-term follow-up of snakebites.
